# Improving linkage to HIV care following a reactive HIV self-testing result among men in KwaZulu-Natal, South Africa

**DOI:** 10.1186/s12913-024-10981-6

**Published:** 2024-04-30

**Authors:** Mbuzeleni Hlongwa, Edward Nicol

**Affiliations:** 1 Public Health, Societies and Belonging, Human Sciences Research Council, Pretoria, South Africa; 2 School of Nursing and Public Health Medicine, University of KwaZulu-Natal, Durban, South Africa; 3 Burden of Disease Research Unit, South African Medical Research Council, Cape Town, South Africa; 4 Division of Health Systems and Public Health, Stellenbosch University, Cape Town, South Africa

**Keywords:** HIV self-testing, Linkage to care, Men, South Africa

## Abstract

**Background:**

Despite the many interventions that have been implemented in sub-Saharan Africa to improve the uptake of HIV testing and antiretroviral (ART) initiation services, the rates at which men are tested for HIV and initiated on ART have remained consistently lower compared to those for women. We aim to investigate barriers and facilitators for linkage to care following HIVST positive results among men aged between 18 and 49 years, and use these findings to design an intervention to improve linkage to care among men in a high-HIV prevalent district in KwaZulu-Natal province, South Africa.

**Methods:**

This multi-method study will be conducted over 24 months in eight purposively selected HIV testing and treatment facilities from December 2023 to November 2025. For the quantitative component, a sample of 197 HIV positive men aged 18–49 years old who link to care after HIV self-test (HIVST) will be recruited into the study. HIVST kits will be distributed to a minimum of 3000 men attending community services through mobile clinics that are supported by the Health Systems Trust, at different service delivery points, including schools, taxi ranks and other hotspots. The qualitative component will consist of in-depth interviews (IDIs) with 15 HIVST users and IDIs with 15 key informants. To design and develop acceptable, feasible, effective, and sustainable models for improving linkage to care, three groups of HIVST users (2*positive (*N* = 12) and 1*negative (*N* = 12)) will be purposively select to participate in a design workshop. Chi square tests will be used to identify social and demographic factors associated with linkage, while logistic regression will be used to identify independent factors. Kaplan Meier curves and cox proportional hazard models will be used to identify factors associated with time to event. Content and thematic approaches will be used to analyze the qualitative data.

**Discussion:**

There remains an urgent need for designing and implementing innovative intervention strategies that are convenient and tailored for addressing the needs of men for improving HIV testing and linkage to care at early stages in resource-limited settings, to improve individual health outcomes, reduce transmission from HIV and minimize HIV-related mortality rates. Our proposed study offers several important innovations aimed at improving linkage to care among men. Our study targets men, as they lag the HIV continuum but are also under-researched in public health studies.

## Background

Gender disparities are pervasive throughout the HIV care continuum in sub-Saharan Africa (SSA), with men testing, receiving treatment, and achieving viral suppression at lower rates, compared with women [1–3]. Evidence has shown that men’s access to services at healthcare clinics in SSA is affected by multiple factors, including stigma, privacy concerns, long waiting times, financial barriers, and confidentiality-related fears [4]. These barriers contribute to men’s delayed access to health care services, leading to higher rates of morbidity and mortality among men, compared with women [5–10]. Despite these persistent disparities, little attention has been given to men’s access to HIV testing and care in SSA [11]. It is critical that males diagnosed with HIV receive HIV treatment early, and that they remain on treatment consistently, to enhance their individual health outcomes, minimize HIV transmission, achieve epidemic control, and extend life expectancy.

HIV self-testing (HIVST) offers a new approach to improving men’s HIV testing rates and removing some of the traditional barriers associated with accessing clinic-based HIV testing services by enabling individuals to conduct and interpret their own HIV tests at their convenient time and chosen private space [12–15].

Several interventions have been designed and implemented in SSA to specifically improve HIV testing rates, linkage, and retention in care among men. Community-based HIV testing interventions have made some strides in improving HIV testing uptake among men who may not otherwise have tested [16]. The high acceptability of HIVST strategy has rekindled hope that individuals testing for HIV will continue to increase [13, 17]. HIVST strategy has reached men generally regarded as ‘hard-to-reach’ with HIV services. In South Africa, the HIV prevalence is highest in KwaZulu-Natal province, accounting for 27% among adults aged 15–49 years [18].

Optimum systems for linking HIVST positive individuals to HIV treatment, and evidence on the barriers for linkage to care following HIVST among men, has not yet been established in SSA [12]. Designing interventions to improving linkage to care is critical for improving HIVST program success in engaging men into HIV treatment, given the limited evidence and challenges on linkage to care following HIVST positive results [19]. Findings from this study will help in designing new service delivery intervention(s) to address some of the barriers deterring men from linkage to care by responding to their distinct needs in uMgungundlovu district, KwaZulu-Natal.

### Significance

Despite the many interventions that have been implemented in sub-Saharan Africa to improve the uptake of HIV testing and antiretroviral (ART) initiation services, the rates at which men are tested for HIV and initiated on ART have remained consistently lower compared to those for women [20]. In South Africa, 72% of men had ever-tested for HIV, compared with 81% tested for HIV among women in year 2019 [21]. Among people living with HIV, 78% of men and 89% of women knew their HIV status in South Africa, and 67% of men compared to 72% of women who were diagnosed with HIV were started on ART in 2017, while men reported poor viral load suppression compared to women [18]. These figures are very low against the 95-95-95 targets set for 2030 by The Joint United Nations Program on HIV / AIDS (UNAIDS) [22]. Attracting men remain one of the key priorities of the UNAIDS 95-95-95 strategic program.

There remains an urgent need for designing and implementing innovative intervention strategies that are convenient and tailored for addressing the needs of men for improving HIV testing and linkage to care at early stages in resource-limited settings, to improve individual health outcomes, reduce transmission from HIV and minimize HIV-related mortality rates. Over half of all male HIV-related deaths occur in men who have never started ART [23]. Mortality rates among people living with HIV remain consistently higher among men compared to women. Between the period 2018–2019, the estimated number of deaths related to HIV-and-AIDS was 74 000 people living with HIV (PLHIV), however, 39 000 of these deaths occurred among males, compared to 31 000 deaths occurring among women [21]. Among PLHIV who have started ART, men are 27% more likely to die from HIV-related illnesses compared to women [23]. The higher figures of HIV-and-AIDS-related deaths among men compared to women result from late HIV diagnosis and ART initiation [6, 7, 9]. Late presentation for HIV services among men further contributes to new HIV infections, thereby reversing the gains achieved in addressing the HIV epidemic. Clearly, early HIV testing and effective linkage to care interventions are key for preventing these downstream impacts.

The high acceptability of HIVST strategy suggests that more men will be reached [17]. HIV self-testing strategy is particularly crucial for the HIV care cascade because it addresses several men’s traditional barriers associated with accessing clinic-based HIV testing services, while enabling individuals to test for HIV at their own convenient time where confidentiality concerns are minimized [4, 15]. Men continuously face several distinctive barriers to accessing clinic-level HIV treatment in South Africa, despite ART being widely available and affordable across the country [4]. In South Africa, women have benefited the most from the expansion of ART, while men have consistently lagged behind. Men are not comfortable accessing clinic-based HIV testing and treatment services citing concerns over long waiting times, confidentiality concerns, stigma, unsuitable operating hours, and that clinics are highly gendered, with HIV services predominantly focused on women of reproductive age [4, 11]. Evidently, the HIVST strategy has proved successful in reaching men with undiagnosed HIV and men otherwise regarded as ‘hard-to-reach’ in resource-limited settings.

Another important gap in the literature is understanding the ways in which different barriers deter different groups of men from linking to HIV treatment following an HIVST positive result, to better design linkage to care interventions aimed at improving the rates of men accessing HIV treatment in SSA. While evidence on linkage to care following an HIVST positive result among men is limited in SSA, inconsistent findings among the few published studies have been reported, with some studies reporting high linkage to care results following HIVST positive, while others have reported very low linkage to care rates among men [17, 24–27]. Importantly, reports on HIVST and linkage to care has been mostly focusing on male partners of pregnant women attending antenatal clinic [28, 29]. Some studies included financial incentives to motivate those who HIVST positive to be linked to HIV care [25, 29]. It is important to note that some of the barriers to linkage to care following an HIVST positive result pertain to the lack of effective mechanism for personal referral system for linkage to care for those who have tested HIV positive [19]. HIVST needs acceptable, feasible, effective, and sustainable approaches for improving linkage to care.

Our intervention focuses on linkage to care as part of the HIVST program design, for which little is known in SSA, especially among men. While HIVST has shown high acceptability among men in resource-limited settings, linkage to care following HIVST positive result has been a persistent blind spot in the HIVST program design. The current approach require HIVST positive individuals to visit a healthcare clinic for confirmation of HIV test results, and starting ART if confirmed HIV positive, which re-introduces the several traditional barriers associated with accessing clinic-based HIV treatment services among men [4, 30]. Our approach is focusing on community-based ART initiation following HIVST positive result. Evidence shows that community-based initiation and delivery of ART services are an effective strategy for improving access to HIV treatment in SSA, as it addresses several distinctive barriers associated with accessing HIV treatment from clinic settings [31–34].

Linkage to care following HIVST positive result among men is a critically important part of the HIVST program. However, optimum systems for improving linkage to care following an HIVST positive result among men are limited in SSA. This study aims to investigate barriers and facilitators for linkage to care following HIVST positive results among men aged between 18 and 49 years, and use these findings to design an intervention to improve linkage to care among men. These will be achieved through the following objectives: (a) to determine linkage to care rates following HIVST positive among men; (b) to determine the barriers and facilitators for linkage to care following an HIVST positive result among men in uMgungundlovu district; and (c) to design an intervention for improving linkage to care following an HIVST positive result among men.

## Methods

### Theoretical framework

The Socio-Ecological Model (SEM) will be used as the conceptual basis underpinning this study. The SEM recognizes that human behaviour is influenced by a variety of circumstances at intrapersonal, interpersonal, community, and societal levels [35]. Using the SEM as a conceptual framework will enable this study to implement a multilevel approach to examine how individual, interpersonal, and institutional factors influence linkage to care following HIVST positive result. We anticipate understanding factors enabling and deterring men’s link to HIV care following HIVST positive, including behaviors, attitudes and structural barriers. Understanding the factors that influence attitudes and intention for linkage to care following HIVST positive among men will inform the development of culturally congruent interventions. Programs designed around the constructs of the socio-ecological model can be critical to improving health outcomes by considering the dynamic and cumulative interplay between the various contextual factors. By linking individuals’ health behaviors with their unique life experiences, they help public health program designers deal with the specific obstacles to behaviour change within a population group more effectively [36]. The model does not often include health care services related factors, however. These will be added to the SEM in this study. The SEM is well established in public health and has been used in similar studies [37, 38].

### Preliminary data

Extensive review of evidence on linkage to care following HIVST positive result among men has informed this proposal. We conducted a systematic review to synthesize available published evidence on linkage to HIV care following HIVST positive result among men in sub-Saharan Africa. Results showed that linkage to care following HIVST positive result is subject to several barriers, including financial constraints due to travelling costs, potential long waiting hours at the health care clinics, stigma, discrimination, and privacy concerns associated with attending a health care clinic setting. For example, studies conducted in Zambia, Uganda, Malawi and South Africa indicated that most poor men, even when they may intend to confirm HIVST results and be initiated on ART at the clinic, if tested HIV positive, may not be able to do so due to financial constraints or travelling costs [26, 27, 29, 39]. Rates of men seeking HIVST confirmation and subsequent linkage to care following HIVST positive result were inconsistent. In South Africa, 72% of men with reactive HIVSTs received a confirmatory test, and 95% who were confirmed HIV positive were subsequently linked to HIV care and started on treatment, when financial incentives were included [25]. However, without the incentives, 68% of men who tested HIVST positive were linked to care and started on treatment [25]. In Malawi, secondary distribution of HIVST kits to male partners by women attending antenatal care showed increased linkage to HIV care post HIVST positive, especially when conditional financial incentives were included [28, 29]. While linkage to HIV care after HIVST has been high in some studies, in a study conducted in Malawi aimed at estimating a timely linkage into confirmatory testing and HIV care following HIVST, the authors reported linkage to care after HIVST at 56.3% [17]. Similar findings were observed in Kenya, where 65% of participants who were newly diagnosed with HIV sought confirmatory testing, and 58% of those were linked to care within the three month follow up [40]. A study conducted in South Africa showed that linkage to confirmatory testing (18.1%, 116/640) as well as HIV care (16.0%, 12/75) after unsupervised HIVST was relatively low, despite telephonic reminders and home visits [27]. Lower rates of linkage to care after HIVST were also presented in Kenya, where only 25% (2/8) of men who were tested positive using the HIVST, went for confirmatory testing, and subsequently linked to HIV care [41]. Similarly, only 23% of men who were HIVST positive in Uganda were linked to HIV care in the intervention arm compared with 66.7% in the control arm [26]. Importantly, reports on linkage to care following HIVST reactive result have mostly focused on male partners of pregnant women attending antenatal clinic. Some studies included financial incentives to motivate those who were HIVST positive to be linked to HIV care. Given the persistent barriers deterring men from accessing HIV services in health care setting, and the inconsistency of linkage to care rates, these suggest the importance of community-based ART initiation following HIVST reactive result.

### Setting, population and community partners

This study will be conducted in uMgungundlovu district in KwaZulu-Natal, South Africa. The district incorporates rural traditional settlements or farmlands through to informal and peri-urban living. UMgungundlovu district is predominantly rural and poor, with high HIV prevalence (31% overall among 15–49 years; 23% among men 15–49 years), unemployment rate over 30% in the last quarter of 2021 [42–44]. Data from the HIV incidence study conducted in uMgungundlovu in 2017 showed that among men aged 15–19, 20–24, 25–29 and 30–35 the HIV incidence rates were 0.24, 1.18, 2.84 and 1.45 per 100 person-years, respectively [45]. The uMgungundlovu Health District is one of the National Health Insurance pilot sites with 46 fixed clinics and 17 mobile clinics. Given the high HIV prevalence among men, the district offers an excellent setting for implementing interventions aimed at improving linkage to care following HIVST positive result among men. Our study will target men aged between 18 and 49 years, a population at a greater risk for new HIV infections. The inclusion criteria for this study comprise being male, being between the ages of 18–49, have not tested for HIV in the past 12 months, never been/not currently on HIV treatment, and residing in the study area. We will work in close collaboration with existing non-governmental organization and community-based structures, Health Systems Trust, as our main implementing partner given their reputation, expertise, and footprint in the district. The organization supports community HIV testing and treatment in uMgungundlovu district. They have strong relationships with the department of health at provincial, district and facility levels, and the communities in uMgungundlovu district. We will engage closely with Health Systems Trust from design to implementation of our intervention.

### Sampling, data collection and analysis

#### Specific Objective 1

We aimed to estimate the proportion of men who link to care following an HIV positive self-test result. To achieve a sufficient sample size for this estimation, we employed a statistical approach using Stata v15.0. Initially, we calculated that a sample size of 197 HIV positive men would be required to estimate this proportion with a precision of ± 10% and a confidence level of 95%, assuming a non-informative baseline estimate of 50%. However, to achieve this sample size, we needed to screen a larger number of men due to the anticipated prevalence of HIV in the population. With an estimated prevalence of 7%, approximately 3000 men needed to be screened to identify the required number of HIV positive individuals.

Using quantitative surveys of HIVST users, we will assess linkage to care rates based on our intervention model (which would include piloting community-based delivery of HIVSTs to men). We will distribute HIVST kits to a minimum of 3000 men attending community services through mobile clinics that are supported by the Health Systems Trust, at different service delivery points, including schools, taxi ranks and other hotspots. We will also use Coaches (men who are living openly with HIV, healthy and stable on ART, and have received training and supervision on providing peer support) and social networks to distribute HIVST kits. We will use the INSTI HIV Self-Test kits, an oral-fluid based test kit. Each kit will be packaged with a leaflet detailing how to conduct HIVST and interpret results. We will also explain the testing procedure and the referral process to men collecting HIVST kits at the distribution sites. The referral process will consist of the following: first, HIVSTs will be distributed to eligible men, who will then perform HIVST (assisted/unassisted); second, follow up will be conducted by our team, using phone calls, SMSs, WhatsApp, or community outreach (tracing and tracking outcomes will be documented); third, if HIVST positive, men will confirm result with trained health care provider at a mobile clinic; fourth, if confirmed HIV positive, men will be started on treatment. We will collect and capture personal details and background characteristics (age, education, ethnicity, religion, marital status, and employment status) of all HIVST individuals using REDCap, a web-based application designed for management of research data. Treatment status for each participant will be validated against local medical records in the TIER.Net database. We will follow-up HIVST positive participants using SMS and phone call reminders. We will follow up participants for at least three months before an outcome is assigned.

We will export data from REDCap into Stata version 15.0 [46] for analysis, and then eliminate discrepancies and remove duplicates. Data will be de-identified prior to final analysis. We will describe baseline characteristics using summary statistics (median, interquartile range [IQR] and proportions) overall and by participant group (linked vs. not linked). We will measure linkage to care outcomes (Y/N/Unknown). For patients linked to care, the time in months from the first day of the month tested positive to the first day of the month participants reported that they confirmed results and enrolled on ART in a mobile clinic will be recorded. We will do both a ‘time to linkage’ and ‘overall’ linkage measures. HIV diagnosed date will be self-reported by the participants. We will assign study participants an outcome at the end of the study as follows: linked to HIV treatment; not linked to HIV treatment. HIVST positive participants will be given at least three months to link to ART, after which outcomes will be allocated. Chi square tests will be used to identify social and demographic factors associated with ‘overall’ linkage. Logistic regression will be used to identify independent factors. Kaplan Meier curves and cox proportional hazard models will be used to identify factors associated with time to event. Stata v15.0 statistical software will be used for the analysis.

#### Specific objective 2

We will conduct in-depth interviews with the HIVST users. We will purposively select ± 15 HIVST users for in-depth interviews We will also purposively select fifteen key informants for in-depth interviews to get their perspectives. Key informants will include individuals from the Department of Health (DoH) employees (national, KZN provincial and district levels), academics (from South African institutions), and representatives of South African non-governmental organizations (NGOs) working in HIV. Key informants will be selected if they have experience working in HIV in South Africa, specifically in KwaZulu-Natal. We will purposively sample key informants based on their expertise and involvement in the implementation of HIV programmes in uMgungundlovu district, especially focusing on men. Interviews will be conducted in a local language (IsiZulu) and translated and transcribed into English. We will audio-record interviews, and take hand-written field notes during the interviews, with permission from the participants. To minimize any inconsistencies during the data collection period, interview guides will be pretested with at least three participants who will not form part of the actual sample. Each interview is anticipated to last between 60 and 90 min. Interviews will elicit perspectives and experiences regarding the barriers and enabling factors for linkage to care following HIVST positive results.

One independent researcher will translate and transcribe data from interviews, and field notes from local language (IsiZulu) to English. Using NVivo version 11, two independent researchers will conduct thematic data analysis, guided by Richie and Spencer’s framework [47]. The framework outlines six stages for conducting qualitative data analysis, including (a) familiarization with the data through reading all the transcripts and listening to the audio recordings; (b) generating initial codes using an open coding method, where each segment of data relevant to this study’s research objective, was coded; (c) development of a thematic framework extracting key themes from the coded data; (d) application of the thematic framework to all the data; (e) charting of the data, enabling systematic comparisons between data sets; (f) analysis of the charts for patterns and associations between and within each unit of analysis. To ensure rigor and accuracy, comparative data analysis will be conducted by one additional skilled independent member, who will independently read all transcripts to gain understanding of the content and scope of data collected. The outcome of coding will be verified, cross-checked and discussed between the two members. This process will involve thorough discussions on the coding outcome and whether the research question was answered.

#### Specific objective 3

Intervention Refinement: We will facilitate two design workshops with HIVST users to design and develop acceptable, feasible, effective, and sustainable models for improving linkage to care, HIVST messaging and communication. The PI and two research assistants will facilitate the workshops. We will purposively select three groups of HIVST users (2*positive (*N* = 12) and 1*negative (*N* = 12)) to participate in workshop. The group with HIVST positive participants will be comprised of linked men (*N* = 12) and unlinked men (*N* = 12). Each workshop will take place over one day, and will encourage participants to share perspectives, present potential interventions to develop models for improving linkage to care following HIVST positive result. Design workshops will be conducted in a local language (IsiZulu) and translated and transcribed into English. We will audio-record workshop discussions, and take hand-written field notes during the workshops, with permission from the participants.

One researcher will translate and transcribe data from workshops, and field notes from local language (IsiZulu) to English language, and conduct thematic data analysis, using NVivo version 11. We will have thorough discussions within our team on the models developed through the design workshops to see whether our objectives for designing an intervention for improving linkage to care following HIVST positive result were met. We aim to present the findings from the workshops and the developed models to local health care officials, collaborating partners, scientists, and other key stakeholders. We will consider feedback from different stakeholders, brainstorm and deliberate about our initial design to make improvements (Table 1).

**Table 1:** Summary of participants, data collection, and outcomes

Objective	Participants	Data Collection (and Sample Sizes)			Outcomes (see key*)
		Surveys	IDIs/FCDs/Form	Observation	
1	Male participants	Baseline surveys (3000)	IDIs with HIVST users (± 15)		BF, LR
2	Key informants		IDIs with KIs (± 15)		BF
3	Men (design workshops)		Workshops with HIVST positive (linked/unlinked) and negative (3 groups of 12)	Ethnographic observation	DM

*Barriers, facilitators (BF), linkage rates (LR), design model (DM)

### Dissemination of the results

The results of this study will be disseminated through several methods. Presentation to stakeholders in the provinces and districts (including the facilities and clinic communities) where the intervention was conducted. We will organize dissemination briefings in the district and in the provincial capital, open to all interested individuals, including representatives from provincial and district AIDS councils, government departments, NGO and CBO implementing partners, research institutions, academic institutions, funders and other civil society representatives. We will submit this research for publication to a peer-reviewed journal for publication contingent on acceptance. We will also host an internal workshop to synthesize pilot learnings and insights, and will identify implications for program design, policy influence, and future funding. We will prepare and disseminate a report detailing our insights and the implications described above that will be shared with donors, implementers, and other national and international stakeholders.

### Ethical implications

Research ethical approval to conduct this survey was obtained from the UKZN Research Ethics Committee (REC). Future amendments to study procedures and instruments will be conducted in full compliance with the UKZN ethics committees prior to implementation. Permission to conduct the research will be sought from the Provincial and District Health Departments in KwaZulu-Natal. Confidentiality of data will be protected through procedures that comply with Good Clinical Practice guidelines, including password protected data entry instruments and electronic storage. All participants will sign the informed consent forms prior to their enrolment.

## Discussion

Our proposed study offers several important innovations aimed at improving linkage to care among men. First, our study targets men, as they lag the HIV continuum but are also under-researched in public health studies. Heterosexual men in sub-Saharan Africa represent the largest unaddressed HIV service gap [48]. Despite the consistent gender disparities on HIV testing and ART initiation across SSA, the development of comprehensive service delivery interventions which could be more responsive to men have been minimal [49]. Women of reproductive age have been the primary focus of HIV services. While men have come on to the radar in recent years [50], there is still a great deal to do to better understand and serve this group with HIV interventions.

Second, we are going to use lessons from research on the value of safe spaces for men (as an intervention) to improve our design process workshops (a research activity). Settings which create safe spaces has been shown to reduce stigma, promote trust, and improve men’s engagement in interventions [51]. We will create opportunities for men to discuss, share perspectives and critically reflect in a safe and conducive space. By creating a secure and private environment, men are expected to engage freely about the barriers and facilitating factors for linkage to care following an HIV positive result, without fear of being judged. The design workshops offer prospects for both delivering thoughtful health education and HIVST knowledge content among participants. This study will also reflect on men’s own experiences, attitudes and beliefs, challenging entrenched patterns of thinking, and identifying opportunities for new practices related to linkage to care following an HIVST positive result in a structured, non-judgmental, safe and accepting environment.

Finally, our intervention will leverage existing community interventions, instead of designing a new intervention. Community-based initiation and delivery of ART has been shown to be an effective strategy for improving access to HIV treatment in SSA, as it addresses many barriers associated with a clinic setting [31–34]. Usually, individuals who HIVST positive are required to visit a healthcare clinic for confirmation of HIV test results and subsequently start antiretroviral therapy (ART), if confirmed positive. This approach presents the traditional barriers deterring men from accessing HIV treatment services in clinic settings. Our approach will use community-based mobile clinics for linking men who HIVST positive to ART. In our intervention, we will work in close collaboration with existing non-governmental organizations and community-based structures which support with community HIV testing and treatment in uMgungundlovu district. We will work in close collaboration with these organizations from design to implementation of our intervention, given their strong relationships with the department of health at provincial, district and facility levels, and the communities in uMgungundlovu district. While our intervention will be designed and implemented in close collaboration with these community structures, our design will be feasible for implementation with any community-based structures supporting with HIV testing and treatment in the district.

## Data Availability

The datasets used and/or analysed during the current study will be available from the corresponding author on reasonable request.

## Abbreviations

ART: Antiretroviral Therapy
DOH: Department of Health
HIVST: HIV self-test
IQR: interquartile range
KZN: KwaZulu-Natal
NGOs: Non-Governmental Organizations
PLHIV: People living with HIV
SEM: The Socio-Ecological Model
SSA: sub-Saharan Africa
UNAIDS: The Joint United Nations Program on HIV / AIDS
